# The Pockmarked Face of the Comte de Mirabeau

**DOI:** 10.3201/eid3108.AC3108

**Published:** 2025-08

**Authors:** Andreas G. Nerlich, Antonio Perciaccante, Simon T. Donell, Raffaella Bianucci

**Affiliations:** Institute of Legal Medicine, Ludwig-Maximilians-University, Munich, Germany (A.G. Nerlich); Azienda Sanitaria Universitaria Giuliano Isontina Department of Medicine, “San Giovanni di Dio” Hospital, Gorizia, Italy (A. Perciaccante); Université Paris-Saclay, Montigny-le-Bretonneux, France (A. Perciaccante, R. Bianucci); Norwich Medical School, University of East Anglia, Norwich, UK (S.T. Donell)

**Keywords:** smallpox, poxviruses, viruses, vaccination, monkeypox, cowpox, camelpox, 18th Century France, Iconodiagnosis, Joseph Boze, Comte de Mirabeau

**Figure:**
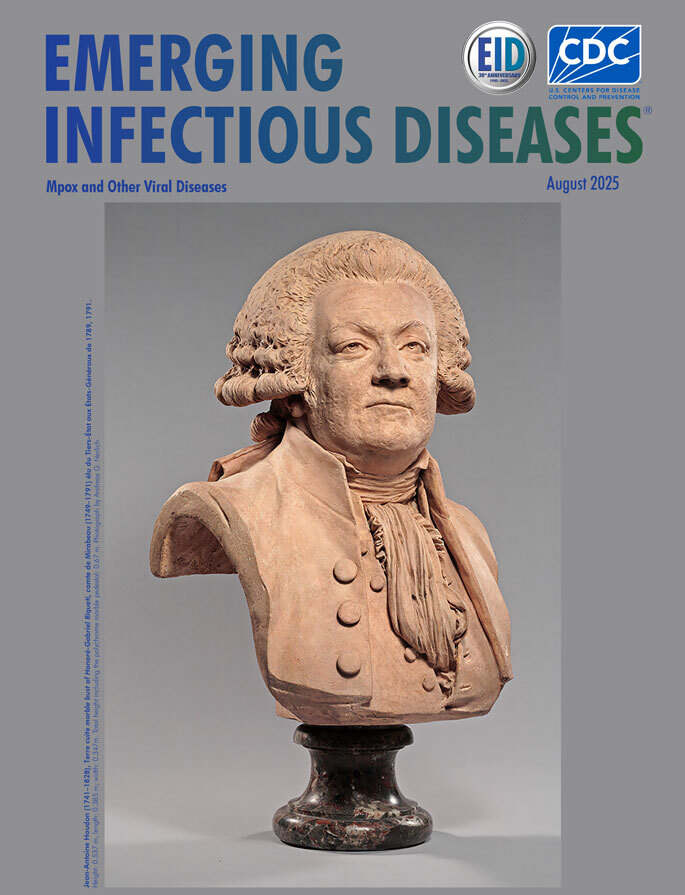
**Jean-Antoine Houdon (1741–1828), Terre cuite marble bust of Honoré-Gabriel Riqueti, comte de Mirabeau (1749–1791), élu du Tiers-État aux États-Généraux de 1789, 1791.** Dimensions: height, 0.537 m; length, 0.385 m; width, 0.347 m. Total height including polychrome marble pedestal, 0.67 m. Louvre-Lens, Galérie du Temps, Lens, France (accession no. RF346). Photograph by Andreas G. Nerlich.

Honoré Gabriel Riqueti, Comte de Mirabeau (1749–1791 CE), was an accomplished French politician, writer, and orator and a distinguished figure in the National Assembly that governed France during the early phases of the French Revolution. Born a member of the prerevolutionary aristocracy, Mirabeau was a moderate and an advocate of constitutional monarchy. He died at age 42 before the revolution reached its radical climax. According to an autopsy, he died of purulent pericarditis and diffuse toxemia (*1*,*2*).

At age 3, Mirabeau suffered a smallpox infection (*1*,*2*). Supportive care was the treatment in that era, and Mirabeau did not develop severe complications, such as blindness (although he had eye problems in later life), cerebral involvement, or sepsis, which generally led to a high (20%–45%) case-fatality rate. He survived the infection but had high concentration of scars on his chin, cheeks, and nose. He was far from the only historical figure to contract smallpox; Mozart, Beethoven, Queen Elizabeth I, Mary Shelley, George Washington, Abraham Lincoln, Queen Mary II of England, Emperor Joseph I of Austria, and Tsar Peter II of Russia all had the disease (*3*).

The mean incubation period for smallpox is 10–12 days. The prodromal phase (2–3 days) is characterized by severe headache, backache, and fever, all beginning abruptly. Enanthema over the tongue, mouth, and oropharynx might precede the rash. The rash has a centrifugal distribution beginning as small, reddish macules, which become papules (2–3 mm) and then vesicles (2–5 mm). The lesions occur first on the face and extremities but gradually cover the body. Pustules (4–6 mm) develop ≈4–7 days after the onset of the rash and remain days to weeks, followed by umbilication and crusting. A second, less pronounced temperature spike might occur 5–8 days after the onset of the rash, especially if the patient has a secondary bacterial infection. The crusts begin separating by the second week of the eruption. Smallpox lesions have a peripheral or centrifugal distribution and are generally all at the same stage of development. Lesions on the palms and soles persist the longest. Death from smallpox is ascribed to sepsis, associated with immune complexes, and to hypotension (*4*,*5*).

Both variola virus (the cause of smallpox) and vaccinia virus (used in smallpox immunization) are associated with ocular complications, including eyelid and conjunctival infection, corneal ulceration, disciform keratitis, iritis, optic neuritis, and blindness (*6*). About 5%–9% smallpox patients developed ocular complications (*7*,*8*).

That Mirabeau had smallpox is confirmed by documentary sources (*1*), and further corroborated by the 1791 terre cuite marble bust by Jean Antoine Houdon (1741–1828) that appears on the cover of this issue of *Emerging Infectious Diseases*. Houdon meticulously reproduced the pockmarks while making the cast of Mirabeau’s face on the day he died (April 3, 1791) (*1*,*2*). Pockmarks were also represented in a hard-paste biscuit porcelain bust by Claude-André Deseine (1740–1823), circa 1791–1792. However, no pockmarks were represented in a 1789 portrait by Louis XVI’s pastellist, Joseph Boze (1745–1826) (Figure). This omission is not uncommon because facial smallpox undoubtedly posed an aesthetic challenge for both literati and painters or sculptors (*9*).

**Figure:**
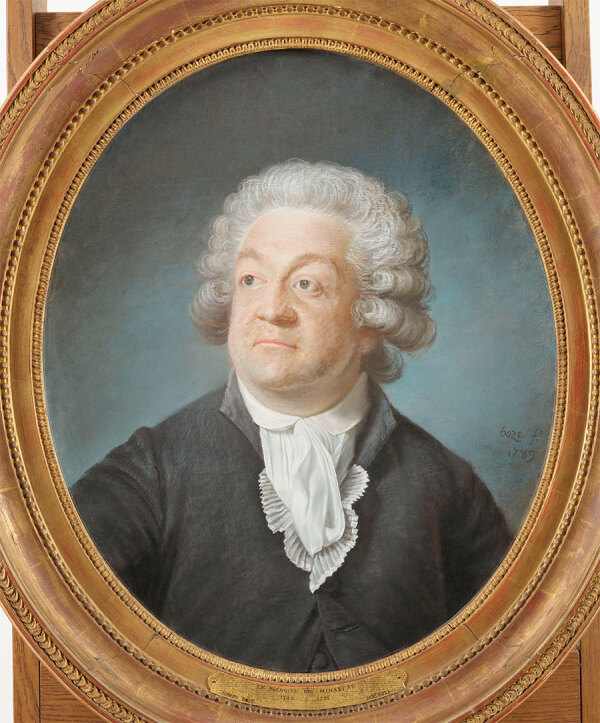
Joseph Boze (1745–1826), Portrait of Honoré Mirabeau, 1789. Pastel on paper, 64.4 cm × 53.5 cm. MV 6032, inv. dessins 1149 RF 6951. Château de Versailles, Versailles, France. Photograph by Franck Raux.

Smallpox was considered a doubly cruel disease, terrifying its victims and leaving survivors permanently disfigured, and was sometimes viewed as a providential collective sin (as written in several elegies) (*9*). Smallpox was not considered “a provisional sin but was historically seen and interpreted as punishment from God, attributed to sin and moral failings, particularly during periods of violent and widespread epidemics” (*9*). Therefore, artistic license was often used when public persons such as Mirabeau were represented (*9*). Although Boze did not represent Mirabeau’s scars in his pastel, he did represent bilateral upper and lower lid ciliary madarosis (*10*,*11*), which manifests in several systemic illnesses, including endocrinopathies, infectious diseases, genetic abnormalities, and some autoimmune disorders (*10*,*11*).

During the 18th Century, ≈50,000–80,000 persons in France and 25,000–30,000 in England died from smallpox each year (*12*–*14*). Those figures also hold true for other countries and regions, which meant smallpox showed comparable mortality rates to plague, at least during outbreaks of the respective diseases (*12*–*14*).

Smallpox was the first infectious disease prevented by targeted (active) immunization, when Edward Jenner used a mild, benign cowpox in 1796 (*15*). However, cowpox might have been used to prevent smallpox in ancient India and, somewhat later, in China (*16*). In Europe, lay variolation was performed in England by Lady Mary Wortley Montague in 1727 (*17*) and by Johan Williamson, who administered smallpox inoculations for ≈3,000 patients during the late 18th Century (*18*); it was also practiced in Germany, in 1767, by Franz Heinrich Meinolf Wilhelm (*19*). However, variolation encountered initial resistance and skepticism from the population. The work of figures such as Voltaire and Catherine II of Russia mobilized the support of influential nobles to overcome hesitation (*20*). Variolation gained renewed popularity in Europe during the 1760s with the rise of the Sutton method (*21*), but that effort came too late for the young Mirabeau, who contracted smallpox in 1751.

After Jenner’s publication (*15*), vaccination was rapidly adopted globally. In France, vaccination was introduced in 1800. Rapid spread of vaccination programs throughout the world took place, and vaccination was strongly promoted in France by Napoleon Bonaparte, preventing troop losses in his army. In addition to massive vaccination campaigns in the 20th Century, development of a strategy involving surveillance and containment effectively led to the eradication of smallpox by 1980 (*22*). Without a natural reservoir, variola virus has since existed only in laboratories; indeed, the last case of smallpox resulted from infection acquired in a laboratory in the United Kingdom in 1978. Today, only the United States and Russia retain variola virus isolates (*23*,*24*).

Despite smallpox eradication, the threat from related viruses remains, and a very low probability of an accidental smallpox virus release exists; such an event could have serious consequences for modern populations, which largely lack immunity. The basic reproduction number (the average number of secondary infections generated by each infected person) for smallpox in contemporary populations has been estimated at 3–6 (*25*).

A further potential issue is the risk for outbreaks or pandemics caused by other orthopoxviruses that can be transmitted to humans, such as camelpox, cowpox, and monkeypox virus. The ongoing international outbreak of monkeypox virus has led to a declaration of a public health emergency by the World Health Organization (*26*). Continued global circulation poses a risk for spillover into new zoonotic reservoirs, which would make managing the virus more difficult. Strict global regulation and cooperation is needed to prevent and control such threats.
